# A retrospective analysis of medications associated with pityriasis rosea reported in the FDA adverse events reporting system

**DOI:** 10.1007/s00403-024-03763-x

**Published:** 2025-01-13

**Authors:** Kayla D. Mashoudy, Joselyn Ye-Tay, Keyvan Nouri

**Affiliations:** 1 Dr. Phillip Frost Department of Dermatology and Cutaneous Surgery, University of Miami Miller School of Medicine, 1150 NW 14th Street, Miami, FL 33136 USA; 2 Department of Pediatrics, Nicklaus Children’s Hospital, 3100 SW 62nd Ave, Miami, FL 33155 USA

**Keywords:** Pityriasis rosea, Drug-induced, FDA adverse events, TNF inhibitors, ACE inhibitors, Biologics, Pharmacovigilance, Adverse drug reactions, Dermatology, Epidemiology

## Abstract

Pityriasis rosea (PR) is an acute exanthematous disease with an uncertain physiopathology, increasingly recognized as potentially drug induced. This study aims to investigate medication triggers associated with PR by analyzing cases reported in the FDA Adverse Event Reporting System (FAERS) database. A retrospective review of 343 PR cases reported in the FAERS database from January 1, 1998, to March 31, 2024, was conducted. Reporting odds ratios (ROR) were calculated to assess associations between PR and specific drug classes, including tumor necrosis factor (TNF) inhibitors and angiotensin-converting enzyme (ACE) inhibitors. Logistic regression analysis evaluated the influence of factors such as sex, age group, and seriousness of outcomes on the occurrence of PR. Females represented 56.3% of cases and the 18–64 age group comprised 55.4% of cases. TNF inhibitors were significantly associated with PR (ROR = 4.1881 [3.1970–5.4865], *P* < 0.0001), particularly infliximab (ROR = 6.5284 [3.9523–10.7837], *P* < 0.0001), etanercept (ROR = 3.4921 [2.2873–5.3315], *P* < 0.0001), and adalimumab (ROR = 3.086 [2.0213–4.7115], *P* < 0.0001). ACE inhibitors were also associated with PR (ROR = 9.9808 [6.0423–16.4864], *P* < 0.0001), with higher odds in older patients (OR 14.08 [4.2–47.2], *P* < 0.0001) and those reporting serious outcomes (OR 9.53 [1.24–72.99], *P* = 0.03). Based on the FAERS, there has been a consistent rise in PR cases, with TNF inhibitors and ACE inhibitors being associated medication classes tied to PR. Given the limited literature on drug-related triggers and patient demographics, we aimed to highlight the characteristics of PR cases that could enhance awareness and inform better clinical outcomes for affected patients.

## Introduction

Pityriasis rosea (PR) is an acute exanthematous disease manifesting as a papulosquamous eruption commonly affecting adolescents and young adults [1]. It is typically characterized by a primary herald patch followed by the development of rose-colored, scaly ovoid patches along Langer’s lines in a “Christmas tree” pattern and the proximal extremities. Pityriasis rosea usually lasts 6–8 weeks and can be difficult to identify with the herald patch often being misdiagnosed as eczema [1, 2]. A prospective survey study found that the total Children’s Dermatology Life Quality Index (CDLQI) scores of children with PR was significantly higher than in children with no active skin issue and those children had significantly lower scores for personal relationships, sleep, and symptoms [3]. Another study highlighted the psychological status of patients with PR and indicated these patients were at higher risk for psychopathology due to uncertain etiology and duration of the recovery period [4].

Currently, the underlying physiopathology of pityriasis rosea is not completely understood. Historically, infectious agents have been implicated in its pathogenesis due to factors like seasonal variation, prodromal symptoms, and case clustering [1, 5–7]. The most common infectious agents reported being human herpes virus (HHV)-6 and HHV-7. However, other infectious etiologies like bacteria, spirochetes, and noninfectious sources like autoimmunity and atopy have been described in in literature as well [1, 7–9]. Over recent years, numerous cases of medication-induced skin eruptions have been reported, involving medications such as captopril, lisinopril, gold, barbiturates, clonidine, D-penicillamine, and nortriptyline [1, 10–12]. More recently, case reports and studies on PR-like eruptions after COVID-19 vaccinations [13–17] and biologic agent therapies like imatinib [18, 19] and tumor necrosis factor (TNF)-alpha inhibitors [20–22] have been published. Nonetheless, causation can be difficult to determine in such cases.

Accordingly, the paucity of literature on medications and triggers that can provoke the development of PR and PR-like eruptions warrants further investigation. The Food and Drug Administration (FDA) created a public database that permits healthcare professionals, consumers, and manufacturers to submit medication adverse event reports. The FDA Adverse Events Reporting System (FAERS) has been utilized as a tool to classify side effects of particular medications and reciprocally, to evaluate drugs that have been associated with certain reactions. The goal of this paper is to highlight associated medications and patient characteristics corresponding to the development of PR cases as recorded in the FAERS.

## Materials and methods

### Data source and collection

Data on adverse events (AEs) with the reaction type “pityriasis rosea (PR)” were extracted from the FAERS Database. All the reports from January 1, 1998, to March 31, 2024, were analyzed. All entries labeled with PR were included, regardless of the reporter’s geographic location or other demographic characteristics. The following criteria were applied:

#### Inclusion criteria

- All reports explicitly indicating PR as an adverse event, based on FAERS coding or descriptive terms.
- Reports from all demographics, including different age groups, genders, and geographic regions.

#### Exclusion criteria

No explicit exclusion criteria were applied due to the anonymized dataset nature and the inability to filter for duplicates or verify completeness.

### Statistical analysis

We performed a descriptive statistical analysis of the clinical and demographic characteristics including sex, age group, reported event year, and most common medications associated. Any cases with missing data regarding sex and age were not included in patient characteristic analyses. A disproportionality analysis was conducted by computing reporting odds ratios (ROR) and corresponding 95% confidence intervals (95% CI) for the association between PR and tumor necrosis factor (TNF) inhibitor class, as well as PR and angiotensin-converting enzyme (ACE) inhibitors overall [23, 24]. ROR was calculated as the ratio of the odds of reporting PR versus all other adverse drug reactions for a given drug, compared with these reporting odds for all other drugs available in the FAERS database. To identify potential associations between PR and medications, logistic regression models were employed. Odds ratios (ORs) with 95% confidence intervals (CIs) were calculated. Specifically, logistic regression was used to calculate the odds ratio (OR) for the occurrence of PR associated with TNF inhibitors and PR associated with ACE inhibitors under different factors, including sex, age group, and seriousness of outcomes. Serious outcomes reported included life-threatening hospitalization, disability, and death. In this study, *P* < 0.05 was considered statistically significant. Confounding factors could not be adjusted because the anonymous nature of entries made it impossible to identify duplicates. A total of 343 PR entries have been recorded in the FAERS since 1998. All statistical analyses were conducted using IBM SPSS^®^ and Microsoft Excel^®^ software platform.

### Primary outcome

The primary outcome of this study was to analyze the frequency of reported cases of PR as an AE in the FAERS database; this was assessed in relation to its demographic characteristics and the frequency of PR cases associated with specific drugs.

## Results

**Fig. 1:**
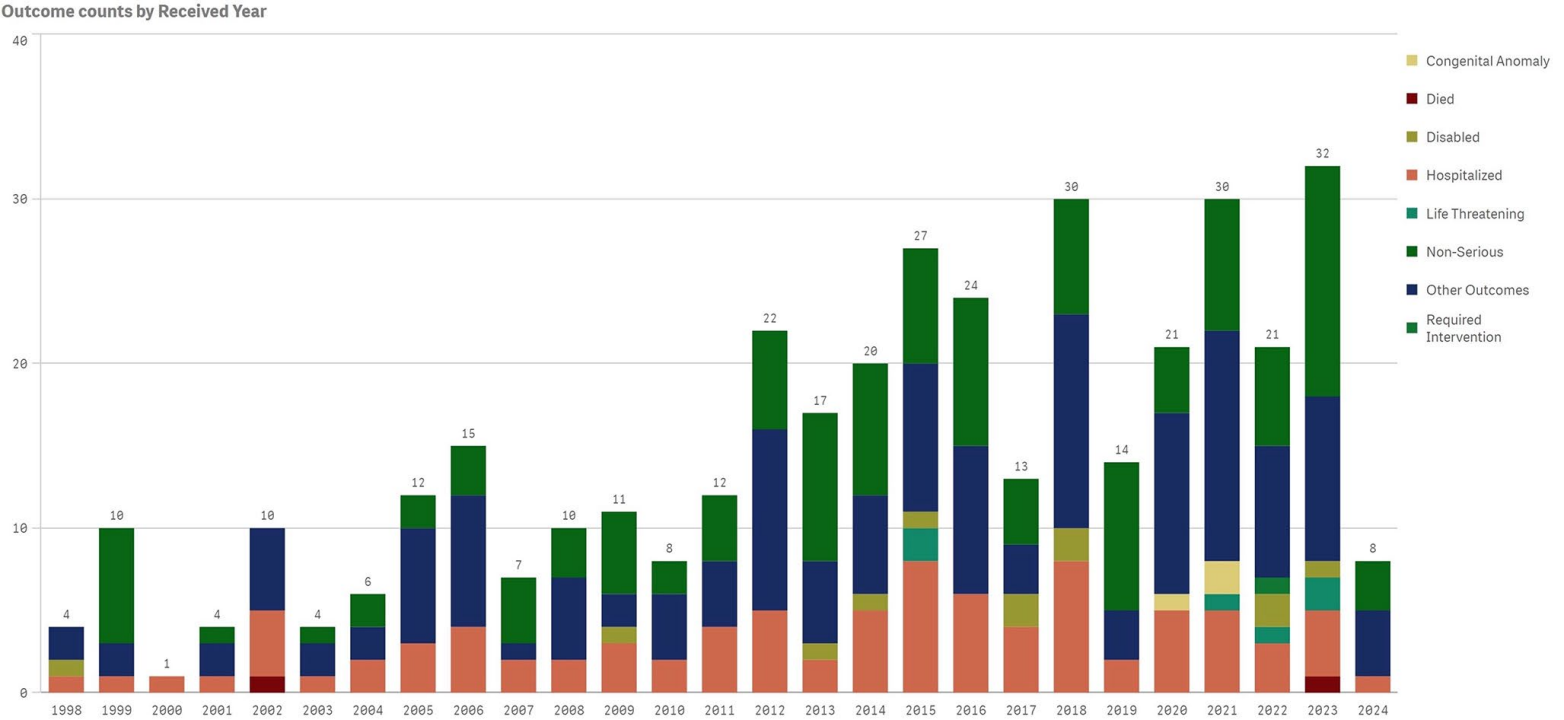
Reports of pityriasis rosea as an adverse event on the FAERS categorized by year of the event and outcome

A total of 343 cases of PR were reported on the FAERS database. The yearly distribution of PR cases reported is illustrated in Fig. 1. The first case of PR documented as a medication adverse event was in 1998. Since then, there has been a consistent rise in reported cases, particularly in recent years. Patient demographics, including sex and age, were also analyzed. Approximately 36.4% (125/343) of PR reports involved males, while 56.3% (193/343) were associated with females (Table 1). As depicted in Table 1, the age group between 18 and 64 years old represented the majority of PR cases, accounting for 55.4% (190/343); followed by individuals older than 65 years old, accounting for 14.0% (48/343).

**Table 1:** Reports of pityriasis rosea as an adverse event on the FAERS by gender and age group

Demographic variables	Total frequency (*n* = 343)	
Gender, n (%)		
Female	193/343	(56.3%)
Male	125/343	(36.4%)
Not specified	25/343	(7.3%)
Age, n (%)		
0-1month	0/343	(0.0%)
2 months-2 years	1/343	(0.3%)
2–11 years	11/343	(3.2%)
12–17 years	14/343	(4.1%)
18–64 years	190/343	(55.4%)
65–85 years	43/343	(12.5%)
more than 85 years old	5/343	(1.5%)
Not specified	79/343	(23.0%)

The 12 most frequent drugs reported to be associated with PR as an adverse event are listed in Table 2. Regarding the top 5 medications reported, 6.7% (23/343) of total PR cases reported on the FAERS database were seen with etanercept, 6.7% (23/343) were with adalimumab, 4.7% (16/343) were with infliximab, 2.9% (10/343) were with dupilumab, and 2.6% (9/343) were with lamotrigine and imatinib, each. It’s noteworthy that among the top 12 medications reported with PR as an adverse event, seven are biologics, including etanercept, adalimumab, infliximab, dupilumab, imatinib, vedolizumab, and natalizumab.

**Fig. 2:**
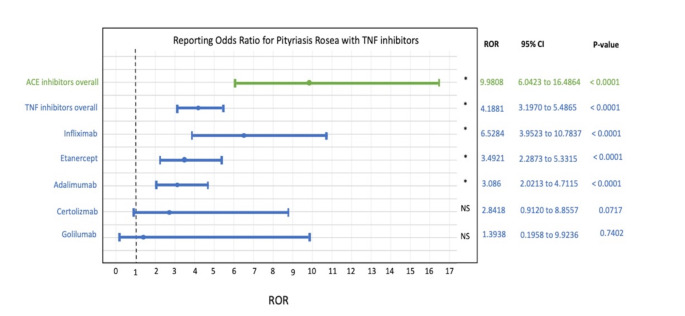
Forest plot showing the reporting odds ratio for pityriasis rosea per given specific drug in the FAERS. ROR indicates reporting odds ratio. NS indicates not significant, *P < 0.001

When drugs were grouped by their mechanism of action, 19.2% (66/343) of cases of PR were associated with TNF inhibitors (adalimumab, etanercept, infliximab, certolizumab, golimumab), while 5.0% (17/343) of PR cases were associated with ACE inhibitors (captopril, cilazapril, enalapril, lisinopril, perindopril, quinapril, ramipril). Based on the full FAERS database, ROR for PR associated with TNF inhibitors and ACE inhibitors were calculated, as shown in Fig. 2. Overall, TNF inhibitors were significantly associated with the occurrence of PR (ROR = 4.1881 [3.1970–5.4865], *P* < 0.0001), as were ACE inhibitors (ROR = 9.9808 [6.0423–16.4864], *P* < 0.0001). Analysis by each TNF inhibitor product was performed, resulting in statistical significance for infliximab (ROR = 6.5284 [3.9523–10.7837], *P* < 0.0001), etanercept (ROR = 3.4921 [2.2873–5.3315], *P* < 0.0001) and adalimumab (ROR = 3.086 [2.0213–4.7115], *P* < 0.0001).

**Table 2:** Most common medications associated with pityriasis rosea reported on the FAERS

MEDICATION	TOTAL FREQUENCY (*N* = 343)	
Etanercept	23/343	6.7%
Adalimumab	23/343	6.7%
Infliximab	16/343	4.7%
Dupilumab	10/343	2.9%
Lamotrigine	9/343	2.6%
Imatinib	9/343	2.6%
Vedolizumab	8/343	2.3%
Natalizumab	7/343	2.0%
Gabapentin	6/343	1.7%
Human Immunoglobulin G	6/343	1.7%
Amlodipine	6/343	1.7%
Terbinafine	6/343	1.7%

**Fig. 3:**
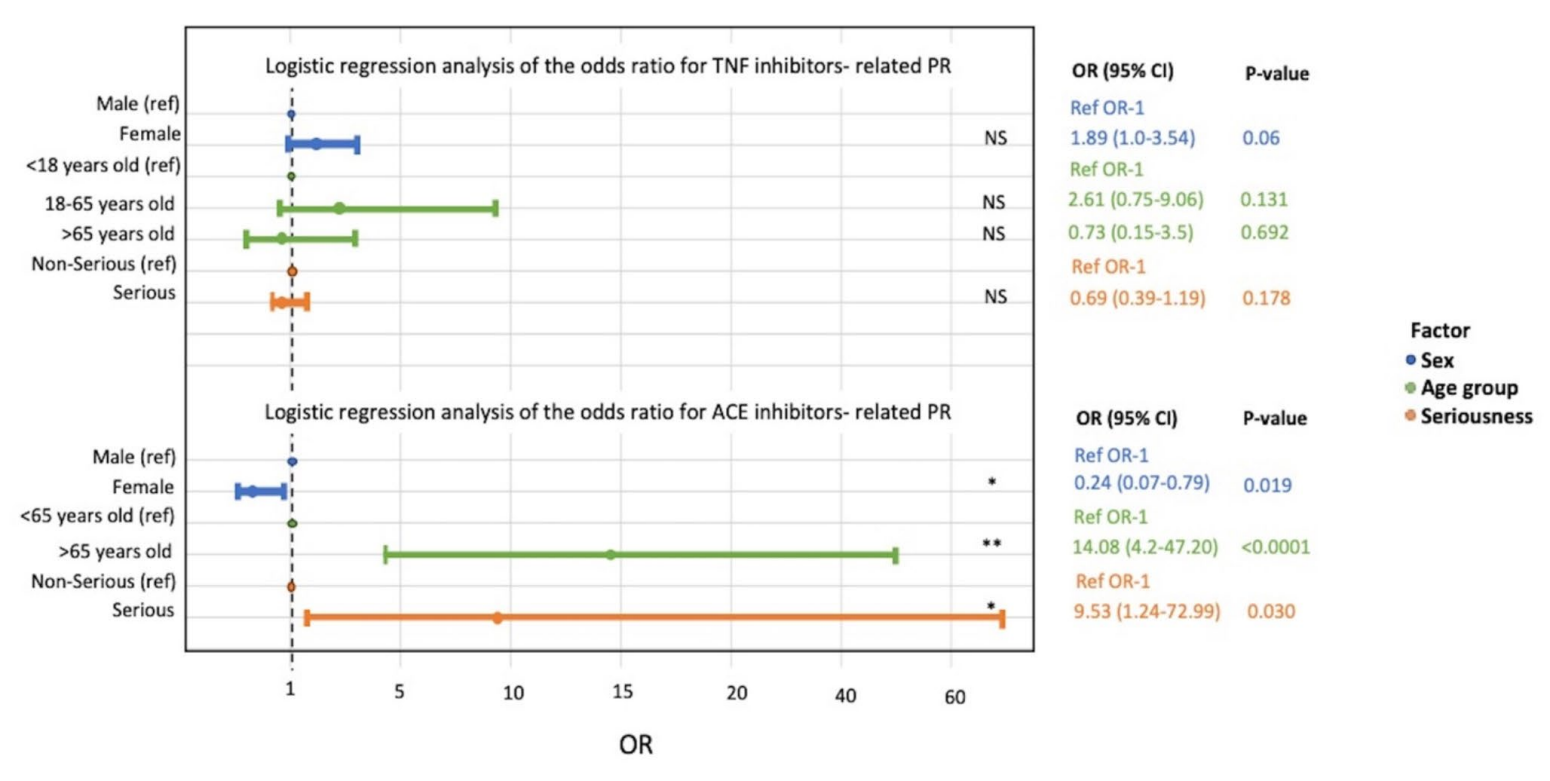
Forest plot of the logistic regression analysis of factors influencing TNF inhibitor-related PR and ACE inhibitor-related PR. The exposure factors considered included sex (blue), age (green), outcome (orange). OR indicates odds ratio. NS indicates not significant, *P < 0.05; **P < 0.001

We further explored factors that might influence the occurrence of TNF inhibitor and ACE inhibitor-related PR by logistic regression analysis based on the total PR cases, as reported in Fig. 3. Among all PR cases, sex, age, and seriousness of outcomes were not influencing factors for TNF inhibitor-associated PR (*P* > 0.05). Females were at about 80% less odds to have reported ACE inhibitor-related PR compared to males (0.24 [0.07–0.79], *P* = 0.019). When comparing age groups, patients older than 65 years old had 14 times higher odds (OR 14.08 [4.2–47.2], *P* < 0.0001) to have reported PR when taking ACE inhibitors. Patients that reported serious outcomes were approximately 10 times more likely (OR 9.53 [1.24–72.99], *P* = 0.03) to have reported PR when taking ACE inhibitors, compared to patients reporting non-serious outcomes.

## Discussion

We present the first comprehensive overview of medications and patient characteristics related to pityriasis rosea as an adverse event reported in the FAERS database. Of the over 28 million side effects recorded in the database, only 0.00001% were PR cases, suggesting a comparably low incidence rate. Although the overall trend of reported PR cases has been steadily increasing, the approximate yearly incidence in the FDA database has stayed considerably lower than epidemiological estimates of PR incidence being 0.5–2% [1, 25, 26]. It’s worth mentioning that data collection for the year 2024 is ongoing but not yet finalized with information gathered only up to March, representing only a three-month period. Given that up to 2% of the general population experience PR at some period in their lives, examining these epidemiological trends and the proportion of cases associated with common medications is noteworthy, especially since the frequency of drug-related PR seems to be underreported [10].

Although the underlying mechanisms and cytokine profile of PR are still poorly elucidated, peaks of immunological research over the past 40 years have strived to identify inflammatory mediators and inciting triggers [27]. Several studies have implicated cell-mediated immunity in the pathogenesis of PR. In 1985, Aiba and Tagami found immunohistological evidence of cellular immune reactions in lesional skin, including increased mononuclear cells, active CD4 + T-helper cells, and Langerhans cells [28]. Sugiura et al. later showed that the increase in Langerhans cells and the T-helper to T-suppressor/cytotoxic ratio correlated with disease stage and severity [29]. Baker et al. [30] and Neoh et al. [31] both confirmed and expanded on these findings, showing increased staining for markers of T cells and Langerhans cells, but not for B cells or natural killer (NK) cells. Other studies by Bos and colleagues [32–34] described the distribution and types of immune cells in PR, further supporting the role of cell-mediated immunity. Additionally, data from papers investigating the sera of PR patients corroborate these findings. Namely, Drago et al. found increased levels of interferon alpha, interleukin 17, and vascular endothelial growth factor [35]; and Gangemi et al., in two separate studies, reported higher levels of fractalkine [36] and interleukin 22 in PR patients [37]. All these studies outline characteristic immune responses to viruses and are cited by several authors as indirect evidence supporting the proposed viral etiology of PR [31, 38].

Medications have also been suggested to play a role in the active immunological response in PR. Specifically, Rajpara et al. reported an interesting case of PR occurring in a patient using the TNF-alpha inhibitor adalimumab for rheumatoid arthritis [20]. The authors suggested that the downregulation of Th1 immune responses from TNF-alpha blockade may have indirectly induced PR by facilitating a viral infection or reactivation. Alternatively, PR could have resulted from an immunological reaction to anti-TNF-alpha antibodies [20]. Similar cases have been noted with other TNF-alpha inhibitors like etanercept [22] and infliximab [21]. Additionally, various immunomodulating drugs like rituximab [39] and imatinib mesylate [40] among others [10] have been reported to cause PR or PR-like skin rashes, though the mechanisms are largely unknown due to the anecdotal essence of these papers. There is no definitive evidence supporting any specific immunological or non-immunological process, and the discussion remains open [41, 42].

Our large cohort study contributes to the growing data on biologics, specifically TNF-alpha inhibitors, as an inciting drug class for PR with the top 3 most involved medications being etanercept, adalimumab, and infliximab. These 3 drugs separately and TNF inhibitors overall as a drug class were significantly associated with the development of PR as compared to PR occurrence with all other medicinal products in the database. Since these biologics have become widely used across a variety of diseases and more than two decades have passed since their introduction into the U.S. market [43], it is likely that more patients are starting to come forward with their side effects. Although TNF-alpha inhibitors caused the highest number of PR, their rate of PR was comparatively low to their various other reported adverse effects in the FAERS database. Indeed, the most common immune-mediated paradoxical cutaneous side effect of anti-TNF agents is the exacerbation or new outbreak of psoriatic skin lesions, which disproportionately involves palmoplantar pustular psoriasis [44, 45]. Nonetheless, heightened awareness of PR as a potential side effect, even at low rates, can enhance preparedness and patient counseling when using such common immune-mediating treatments.

Interestingly, despite responsibility having been more frequently assigned to ACE inhibitors for PR eruptions [46–50], our results showed that ACE inhibitors accounted for only a small fraction (4.96%) of reported cases. Being male, older than 65 years old, and reporting serious outcomes were all significantly associated with developing ACE inhibitor-related PR. According to Wilkin’s hypothesis [46–48], ACE inhibitors might raise plasma and tissue levels of kinins, which have pro-inflammatory effects linked to various cutaneous adverse reactions, including pityriasis rosea-like eruptions. The role of ACE inhibitors in these reactions warrants further investigation and highlights similarities in the pro-inflammatory pathways affecting the skin.

Females were also reported to develop PR as an adverse event markedly more often than males, which align with most findings in the literature on sex differences [8, 25, 51, 52]. However, some studies have reported a male predominance [53] or a slight sex variance favoring males [54]. Our large data set analysis contributes to this body of literature. Given this heterogeneity, future research should control for drug use discrepancies between sexes to better identify the patient profile at higher risk for PR.

Age groups with the largest number of cases were 18–64 years old followed by individuals older than 65 years old. Prior studies have reported similar age demographics showing greatest prevalence between the ages of 10 and 39 years [25] and less than the age of 30 years [51]. Another prospective study of 214 patients found peak incidence to be in the 20–24 age group [53]. Our data showed that male gender and older people were more likely to report ACE inhibitor-related PR cases as compared to females and younger people. Other investigations of medication-related PR show some unpredictability in both patient age and sex, varying from the 3rd decade upwards [2, 10, 46, 48, 50] and no consistent predominance, respectively [2, 10, 46, 48, 50]. However, a 3-year prospective study found the mean age to be 68.6 years, with a clear male predominance (6 males to 2 females) [10]. The primary responsible drugs were angiotensin-converting enzyme (ACE) inhibitors, either alone or in combination with hydrochlorothiazide [10]. Given that we analyzed cases of medication-induced PR, often involving treatments for immune-mediated disorders, it is unsurprising that we observed few pediatric cases in this subset. For a majority of pediatric conditions, biologics are not currently utilized as therapeutic options [55], especially since safety concerns for the chronic use of human TNF inhibitors like adalimumab, etanercept, and infliximab are significant [56]. In fact, a cross-sectional analysis of pediatric FAERS individual case safety reports (ICSRs) showed that the annual proportion of pediatric ICSRs in the FAERS from 2010 to 2020 ranged between 2.8% and 3.7% of the total number of ICSRs [57]. These represent a minority of FAERS reports but have unique attributes including products and types of AEs reported relative to adult ICSRs. Reporting patterns also vary within pediatric subgroups, which highlights the need for unique considerations for pediatric pharmacovigilance [57]. Moreover, PR cases are frequently documented in teenagers and young adults, potentially resulting in underreporting within this database. Mechanistic variances between drug-induced PR and idiopathic cases could also exist. Considering the myriad of potential confounding factors, additional controlled studies are necessary to establish whether age is an independent predictor of PR.

## Limitations

The FAERS database is a useful tool that facilitates various activities such as identifying potential safety concerns that might be related to a marketed product, assessing a manufacturer’s compliance to reporting regulations, and addressing external inquiries for information. It includes reports submitted by physicians, pharmaceutical sponsors, and patients. However, there is no certainty that the reported event was due to a specific product. Because reporting to FAERS is voluntary, the data may be incomplete compared to real population data. FAERS reporting exhibits biases related to its newsworthiness and legal and scientific variables, as it does not differentiate between PR and PR-like eruptions. Additionally, due to the retrospective nature of the FAERS, a causal relationship between medications and PR cannot be definitively established. Results represent associations only. Furthermore, PR is diagnosed clinically without pathological confirmation, which can lead to misdiagnosis. The anonymized nature of the reports prevents the identification and removal of duplicate entries, potentially resulting in multiple reports of the same case from different sources. Finally, pediatric reports represent a minority of FAERS reports.

## Data Availability

The data that support the findings of this study are available from the corresponding author upon reasonable request.
